# Lysosomal Acid Lipase Activity Is Reduced Both in Cryptogenic Cirrhosis and in Cirrhosis of Known Etiology

**DOI:** 10.1371/journal.pone.0156113

**Published:** 2016-05-24

**Authors:** Umberto Vespasiani-Gentilucci, Paolo Gallo, Fiorella Piemonte, Elisabetta Riva, Aldostefano Porcari, Ferruccio Vorini, Giulia Tozzi, Livia Piccioni, Giovanni Galati, Antonio De Vincentis, Simone Carotti, Sergio Morini, Jessica D’Amico, Silvia Angeletti, Claudio Pedone, Antonio Picardi

**Affiliations:** 1 Internal Medicine and Hepatology Unit, University Campus Bio-Medico, Rome, Italy; 2 Unit of Neuromuscolar and Neurodegenerative Diseases, Children’s Hospital and Research Institute “Bambino Gesù”, Rome, Italy; 3 Virology Unit, University Campus Bio-Medico, Rome, Italy; 4 Laboratory of Microscopic and Ultrastructural Anatomy, CIR, University Campus Bio-Medico, Rome, Italy; 5 Clinical Pathology and Microbiology Laboratory, University Campus Bio-Medico, Rome, Italy; 6 Area of Geriatrics, University Campus Bio-Medico, Rome, Italy; University of Navarra School of Medicine and Center for Applied Medical Research (CIMA), SPAIN

## Abstract

Lysosomal acid lipase deficiency (LAL-d) is a rare autosomal recessive disease in which LAL activity is almost absent, with consequent massive microvesicular steatosis evolving to cirrhosis and liver failure. We aimed to determine LAL-activity, and to investigate the most common single nucleotide polymorphism (SNP) affecting the LIPA gene and responsible for 50–70% of LAL-d cases (rs116928232 c.894G>A), in patients with cryptogenic cirrhosis. Sixty-three patients with cryptogenic cirrhosis, 88 cirrhotics of known etiology, and 97 healthy subjects were enrolled. LAL-activity was determined in dried-blood-spot (DBS). The c.894G>A mutation was analyzed by pyrosequencing method in SNP mode. LAL-activity was severely reduced in patients with cryptogenic cirrhosis with respect to healthy subjects [0.62 (0.44–0.86) *Vs* 0.96 (0.75–1.25) nmol/spot/h, p<0.001)], but it was also reduced in known-etiology cirrhotics [0.54 (0.42–0.79) nmol/spot/h, p<0.001 *Vs* healthy subjects; p = 0.5 *Vs* cryptogenic cirrhotics]. Fourteen percent of cryptogenic cirrhotics and 20% of known-etiology cirrhotics showed a LAL-activity in the range of heterozygous carriers of LIPA gene mutations (0.15–0.40 nmol/spot/h). However, none of the subjects with reduced LAL-activity carried the c.894G>A SNP except for one patient with HCV cirrhosis. By multivariate analysis, LAL-activity was not associated with age, sex, liver enzymes, liver function or lipid parameters, while it was independently associated with white blood cell (β = 0.2; p<0.01) and platelet (β = 0.4; p<0.001) counts and with the condition of cirrhosis (β = -0.2; p = 0.04).

**Conclusion:**

Liver cirrhosis is characterized by a severe acquired reduction of LAL-activity, the precise causes and consequences of which need to be further addressed. DBS-determined lysosomal enzyme activities seem to be affected by white blood cell and platelet counts, and the specificity of these tests can be reduced when applied to determined populations, such as cirrhotics.

## Introduction

Lysosomal acid lipase (LAL) plays a key role in lipid metabolism. Indeed, after low-density lipoproteins are endocytosed into the cells and transported to the lysosomes, LAL catalyzes the hydrolysis of cholesteryl esters and triglycerides, and the resulting free cholesterol and fatty acids are critical mediators in cellular cholesterol homeostasis [1].

LAL deficiency (LAL-d), i.e., Wolman disease and cholesteryl ester storage disease (CESD), is an autosomal recessive disease in which patients are homozygous or compound heterozygous for LIPA gene mutations. The most common inherited defect affecting the LIPA gene is the exon 8 splice site mutation, E8SJM (c.894G>A), which is found in 50–70% of LAL-d patients [2,3]. LAL-d is characterized by lysosomal accumulation of cholesteryl esters and triglycerides in the liver, spleen, lymph nodes and other tissues, consistent with the broad expression of LAL [4].

Massive microvesicular steatosis evolving to cirrhosis and/or liver failure is a hallmark feature of both Wolman disease and CESD [5]: indeed, hepatomegaly and elevated liver enzymes from unknown etiology are among the key elements in the algorithm which should direct to LAL-d screening [1]. Unlike nonalcoholic fatty liver disease (NAFLD) and nonalcoholic steatohepatitis (NASH), which are typically associated with metabolic syndrome, hepatic steatosis depending on LAL-d is usually not observed in a context of obesity and insulin resistance [5], and LAL-d should be particularly suspected in non-obese patients with steatosis or cryptogenic cirrhosis, especially if presenting abnormalities in cholesterol metabolism. However, very recently, a reduction in LAL activity was described also in a cohort of adult NAFLD patients, being more severe in the subgroup with NASH than in the remaining NAFLD population, thus suggesting that an impaired LAL function may contribute to intracellular fatty acid accumulation and damage progression also in the context of NAFLD [6]. Even more interestingly, LAL replacement therapy with Sebelipase alfa in patients with severe LAL-d has been demonstrated to dramatically reduce hepatic fat content as assessed by means of magnetic resonance imaging [7], and Sebelipase alfa treatment has been recently approved by the Food and Drug Administration and the European Medicines Agency for use in these patients.

Recently, a new assay for determining LAL activity in dried blood spot (DBS) has been developed and found to differentiate clearly between normal controls, carriers and affected patients [8]. This test measures lipase activity of blood in presence of Lalistat-2, which is a highly specific LAL inhibitor. In comparison to the determination of lysosomal enzyme activity in leukocytes, analysis on DBS has the great advantages of significantly simplifying the process and reducing the costs [9]. However, since most if not all the enzymatic activity dosed in DBS is thought to derive from leukocytes [9,10], alterations of the white blood cell count could theoretically affect the results and should be taken into account.

To the best of our knowledge, to date, there have been no studies evaluating LAL activity in patients with cryptogenic cirrhosis, which, in the majority of cases, is considered the result of long-lasting NASH [11,12]. Indeed, only one latest study reported a reduced LAL function in advanced liver disease [13]; however, among the 22 patients included in that study, only 4 were affected by cryptogenic cirrhosis. Therefore, the present study was aimed at: 1) evaluating DBS-determined LAL activity in a group of patients with cryptogenic cirrhosis, and compare results with those obtained in a group of patients with cirrhosis of known etiology and in a group of age- and sex-matched controls; 2) investigating all subjects with a reduced LAL activity for the E8SJM mutation; 3) evaluating epidemiologic, clinical and biochemical parameters associated with LAL function in healthy subjects and in cirrhotic patients.

## Patients and Methods

### Patients

Consecutive patients attending the Hepatology Unit of the University Hospital Campus Bio-Medico of Rome (both as outpatients and as inpatients) between January 2013 and December 2015 and receiving the diagnosis of cryptogenic cirrhosis were enrolled in the present study. More specifically, before inviting each of these patients to participate to the study, it was confirmed that there was no history of alcoholic intake >20 gr/day if woman and >30 gr/day if man or of use of drugs known to induce liver damage, and that the following results had been obtained: negative anti-HCV antibodies and HBsAg; antinuclear antibodies (ANA) <1:80 and negative anti-mitochondrial (AMA), anti-smooth muscle (ASMA) and anti-liver and kidney microsomal (anti-LKM) antibodies; normal transferrin saturation and serum levels of ceruloplasmin and alpha-1 antitrypsin.

Two control populations were also included: 1) a group of patients with cirrhosis of known etiology; 2) a group of subjects without clinically-significant liver disease. The first group was recruited among consecutive patients attending the Hepatology Unit of the University Hospital Campus Bio-Medico between January 2014 and December 2015 and receiving the diagnosis of cirrhosis with a well-defined and proven etiology (HCV-, HBV-, alcohol-related, autoimmune, etc…). To note, history/presence of hepatocellular carcinoma (HCC) was considered an exclusion criterion for all cirrhotic patients (cryptogenic and with known etiology). The second control population was recruited among consecutive subjects aged ≥50 years undergoing general medicine company check-ups at the University Hospital Campus Bio-Medico between January 2015 and December 2015. Age ≥50 years was selected in order to render the group comparable to the two populations of cirrhotics. Only subjects with a medical history negative for liver disease, and with normal liver enzymes/function and negative hepatic ultrasound at the check-up examination (only mild steatosis was not considered an exclusion criterion) were invited to participate to the study.

All subjects enrolled in the present study agreed to participate and signed an informed consent. The protocol of the study conformed to the ethical guidelines of the 1975 Declaration of Helsinki and was approved by the Ethics Committee of the University Campus Bio-Medico of Rome.

## Methods

Epidemiological, anthropometric and clinical data were recorded for all subjects. Hepatic enzymes and liver function tests (bilirubin, albumin, INR), if not available within 3 months before enrollment in the study, were repeated. All subjects underwent blood drawing after a 12-hour fast and LAL activity was dosed with dried blood spot (DBS) technique using the inhibitor Lalistat-2. Further venous blood was stored at -80° and, when required (see below), subsequently used for DNA extraction and analysis for E8SJM mutation.

### DBS preparation

The DBS samples were prepared using Whatman paper #903, according to the requirements of the National Committee for Clinical Laboratory Standard Protocol (NCCLS). Ethylene-diamine-tetra acetic acid (EDTA) blood was spotted onto filter paper on the day of venipuncture and allowed drying overnight at room temperature. Samples were stored double-bagged with desiccant at −20°C and analyzed within 1 week of collection.

### Chemicals and reagents

Cardiolipin (sodium salt, bovine), sodium acetate (trihydrate), 4-methylumbelliferone (4-MU), dimethylsulfoxide (DMSO), Mercuric chloride and Triton X-100 were obtained from Sigma Aldrich Company Ltd (Dorset, England). 4-MU palmitate was from Apollo Scientific. Lalistat-2 was supplied by Alexion (Cheschire, Connecticut, USA). A buffer solution was prepared using 0.15 M acetate buffer at pH 4.0, 1.0% Triton X-100. Fourteen ml buffer was retained at 37°C (water bath) and 1.0 ml (0.5%, w/v) cardiolipin in methanol and 400 μl (13.3 mM) 4 MU-palmitate in DMSO were added to give substrate buffer solution. Thirty μM Lalistat-2 was prepared fresh each time by diluting 200 μM Lalistat-2 (in DMSO) with distilled water.

### Lysosomal Acid Lipase assay

The LAL activity was determined by subtracting activity in the inhibited reaction from uninhibited reaction (total lipase) and expressed as nmol/spot/h of 4MU (methylumbelliferone). A 3.2 mm spot was punched into the well of a 96 microtiter plate (Greiner bio-one, Germany) and eluted in 200 μl water for 1 h at room temperature under kind agitation (50 x g). Reactions were performed in duplicate. The reaction mixture contained 40 μl sample and 10 μl of the inhibitor Lalistat-2 (30 μM final). After 10 min pre-incubation, 150 μl substrate buffer was added and the plate incubated for 3 h at 37°C. Reaction was stopped by adding 100 μl (15mM) HgCl_2_. A 0–2.5 nmol/well 4 MU standard curve was built. The fluorescence intensity was measured using a Multimode plate reader EnSpire (Perkin Elmer, USA) (λ excitation = 355 nm, λ emission = 460 nm).

### Analysis for the E8SJM mutation in the LIPA gene

The analysis of the SNP rs116928232 (G>A) was performed by pyrosequencing (PyroMark_Q96 instrument ID; Biotage, Uppsala, Sweden). The amplification and sequencing primers were designed using the software PyroMark PSQ Assay Design v.1.0.6 (Biotage, Uppsala, Sweden), using the genomic sequence including the exon 8 splice junction (NCBI Reference Sequence NG_008194). Genomic DNA (200 ml) was extracted from whole blood using the kit EZ1_DNA blood mini kit (Qiagen Milan, Italy), as instructed by the manufacturer. Five milliliters of the extract were amplified using the specific primers in which the sense oligonucleotide (Fw) was labeled with biotin for subsequent use in pyrosequencing (Fw [BIO] TCT GTG CAA AAC ATG TTA CAC TGG; Rw ATG AAC CCC AAA TGC ACT CC). Twenty μl of the amplified product were subsequently pyrosequenced in SNP mode using the sequence primer (seq CTC CTG GAA TGC CTA C) designed to base-pair upstream of the specific SNP. The sequence analysis was subsequently analyzed through the relative software under the control of the operator.

### Statistics

Data are expressed as mean (standard deviation), median and 25–75% interquartile range or number and percentage, as adequate. Comparisons between the three patient populations were performed by the χ^2^ test for categorical variables, and by the Kruskal-Wallis test, followed by the Mann-Whitney test for post-hoc analysis, for continuous numeric variables. Univariate linear regression analysis was carried out to evaluate the association of different variables with LAL activity. Variables whose association with LAL activity at the univariate analysis showed a p<0.1 were entered the multivariate models, which were used to identify factors independently associated with LAL function. A p<0.05 was considered statistically significant. SPSS software (version 22.00; SPSS Inc., Chicago, IL, USA), was used for statistical analyses.

## Results

### Main characteristics of the study population

Sixty-three patients with cryptogenic cirrhosis, 88 patients with cirrhosis of known etiology and 97 healthy controls were recruited. Among cryptogenic patients, the diagnosis of cirrhosis was clinical (biochemical and ultrasonographic ± upper endoscopic ± transient elastographic findings) in 52 cases, while the other 11 patients had received a histological diagnosis of cirrhosis but not even histology had led to definitive etiologic attribution. In patients with disease of known etiology, the diagnosis of cirrhosis was clinical in 80 cases and histological in the remaining 8 patients. Mean BMI of patients with cryptogenic cirrhosis was 30.9 (SD: 7.8) kg/m^2^, and 43 of them (68%) had type 2 diabetes mellitus, consistent with the notion that the majority of cryptogenic cirrhosis are actually the evolution of NASH cases (11,12). Most cirrhosis of known etiology were HCV-related [49 cases (56%)], while the other were alcoholic [25 cases (28%)], HBV-related [7 cases (8%)], or the result of autoimmune hepatitis [4 cases (5%)] and primary sclerosing cholangitis [3 cases (3%)]. In agreement with inclusion criteria, all healthy controls had normal liver enzymes and negative hepatic ultrasound, excluding mild steatosis which was observed in 33 subjects (34%).

As reported in Table 1, the three study populations were age- and sex-matched. As expected, transaminase levels were significantly higher, while total cholesterol, HDL cholesterol and LDL cholesterol significantly reduced, in the two groups of patients with cirrhosis with respect to healthy controls. Compared to controls, triglyceride levels were not significantly different in patients with cryptogenic cirrhosis, while they were slightly reduced in patients with cirrhosis of known etiology. Consistent with the condition of hypersplenism, which is typical of liver cirrhosis with portal hypertension, the two groups of cirrhotic patients showed significantly reduced white blood cells and platelets with respect to healthy controls. Finally, the two groups of patients with cirrhosis were well-matched in terms of Child-Pugh class distribution, presence of oesophageal varices and mean splenic volume.

**Table 1:** Main characteristics of the study populations, according to subgroups.

	Heathy controls	Cryptogenic cirrhosis	Cirrhosis of known etiology	p
**n.**	97	63	88	-
**Age (years)**	66.0 ± 8.3	68.0 ± 9.7	65.3 ± 10.5	0.3
**Sex (M/F)(%)**	49/48(51%/49%)	32/31(51%/49%)	53/35(60%/40%)	0.3
**AST (IU/L)**	18.2 ± 6.6	39.2 ± 16.9**	44.5 ± 31.9**	<0.001
**ALT (IU/L)**	25.9 ± 8.8	40.9 ± 19.8**	44.8 ± 43.7*^	<0.001
**Total cholesterol (mg/dL)**	203.6 ± 42.5	142.9 ± 36.6**	151.3 ± 44.2**	<0.001
**HDL cholesterol (mg/dL)**	52.2 ± 15.4	39.8 ± 14.8**	46.5 ± 19.1*^	<0.001
**LDL cholesterol (mg/dL)**	126.2 ± 38.9	74.2 ± 31.5**	85.8 ± 32.8**^	<0.001
**Triglycerides (mg/dL)**	126.4 ± 72.8	144.1 ± 68.8	105.4 ± 42.6*^^	0.001
**White blood cells (cells/mm**^**3**^**)**	6440 ± 1654	4855 ± 1559**	5696 ± 2945**	<0.001
**Platelets (cells/mm**^**3**^**)**	238593 ± 25917	115048 ± 51536**	121000 ± 70250**	<0.001
**Child-Pugh class (A/B/C)**	-	38/18/7	50/32/6	0.5
**(%)**		(60%/29%/11%)	(57%/36%/7%)	
**Oesophageal varices (Yes/No)**	-	29/34	38/50	0.7
**(%)**		(46%/54%)	(43%/47%)	
**Splenic volume (cm**^**2**^**)**		71.7 ± 35.7	64.7 ± 30.1	0.2

*p<0.05 and **p<0.01 *Vs* healthy controls; ^p<0.05 and ^^p<0.01 *Vs* cryptogenic cirrhosis

### LAL activity in patients and controls

**Fig 1:**
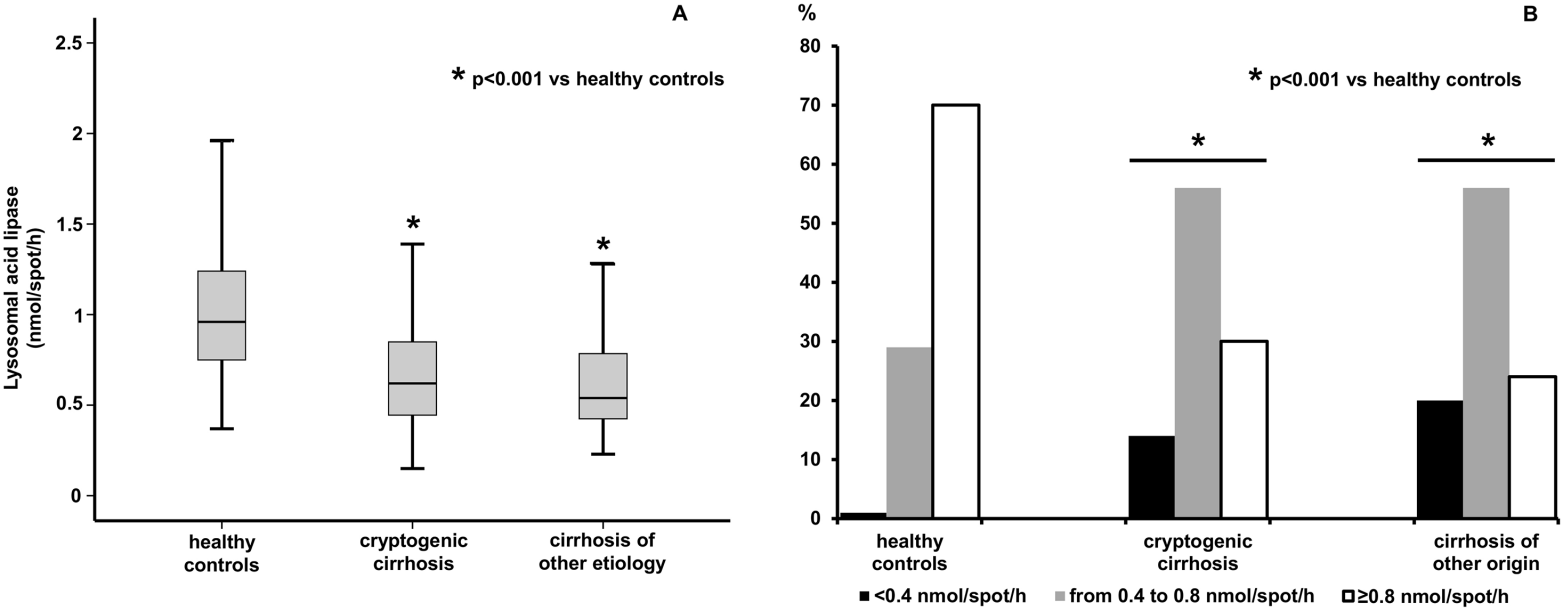
DBS-determined LAL activity in cryptogenic cirrhotics, cirrhotics of known etiology and healthy subjects (Panel A). Percentages of cirrhotic patients and healthy subjects with normal (≥0.8 nmol/spot/h), mildly reduced (range 0.4–0.8 nmol/spot/h), or severely reduced LAL activity (<0.4 nmol/spot/h) (Panel B).

Fig 1), Panel A, shows DBS-determined LAL activity in the two populations of cirrhotics and in healthy controls. Median LAL activity was significantly reduced both in patients with cryptogenic cirrhosis [0.62 nmol/spot/h (0.44–0.86)] and in cirrhotics of known etiology [0.54 nmol/spot/h (0.42–0.79)] with respect to that in controls [0.96 nmol/spot/h (0.75–1.25)] -p<0.001 for both comparisons-, while the difference between the two groups of cirrhotics was not significant (p = 0.5). Indeed, the majority of cirrhotic patients (74% considering altogether the cirrhotic population) evidenced at least a mild reduction of LAL activity (<0.8 nmol/spot/h) (Fig 1, Panel B). Moreover, while none of the subjects analyzed in this study had a DBS-determined LAL activity compatible with the diagnosis of Wolman or CESD, i.e. <0.03 nmol/spot/h [8], 14% of cryptogenic cirrhotics and 20% of cirrhotics of known etiology, but only 1 (1%) of controls, showed a LAL activity in the range which has been described in heterozygous carriers of LIPA gene mutations, i.e., in the range 0.15–0.40 nmol/spot/h [8] (Fig 1, Panel B).

As reported in Table 2, at univariate analyses, neither in the three study populations analyzed separately nor in cirrhotic patients evaluated as a whole, LAL activity was significantly associated with age and transaminase levels. Concerning blood lipid parameters, significant direct associations with LAL function were registered only for triglycerides in healthy controls and for total cholesterol in cirrhotics of known etiology. Notably, excluding the lack of association between LAL activity and white blood cells in healthy controls, strongly significant direct associations were observed between LAL function and white blood cell and platelet counts in the three study populations and in cirrhotic patients as a whole. In patients with cirrhosis of known etiology, LAL activity was inversely associated with splenic volume, and inverse associations with both splenic volume and the presence of oesophageal varices were registered also in the cirrhotic population evaluated as a whole. Conversely, LAL activity was not associated with liver function as determined with Child-Pugh class in any of the cirrhotic groups. Excluding the subgroup of cryptogenic cirrhotics, multivariate analyses confirmed the independent associations between LAL function and white blood cell and platelet numbers observed at the univariate analyses, and the direct association between triglycerides and LAL activity registered in controls. Moreover, a direct independent association between male sex and LAL function was observed only in the group of cirrhotics of known etiology.

**Table 2:** Univariate and multivariate linear regression analysis to LAL activity according to the three study subgroups and in the cirrhotic population as a whole.

**HEALTHY CONTROLS**							
**Univariate**				**Multivariate**			
	**β**	**95% CI for B**	**p**		**β**	**95% CI for B**	**p**
**Age (years)**	-0.02	-0.01–0.01	0.88	**Age**	-	-	-
**Sex (F = 0; M = 1)**	-0.11	-0.25–0.08	0.29	**Sex**	-	-	-
**AST (IU/L)**	-0.04	-0.02–0.01	0.67	**AST**	-	-	-
**ALT (IU/L)**	0.002	-0.01–0.01	0.98	**ALT**	-	-	-
**Total Cholesterol (mg/dL)**	-0.03	-0.002–0.002	0.80	**Total Cholesterol**	-	-	-
**LDL Cholesterol (mg/dL)**	-0.10	-0,003–0.001	0.33	**LDL Cholesterol**	-	-	-
**HDL Cholesterol (mg/dL)**	-0.04	-0.006–0.004	0.72	**HDL Cholesterol**	-	-	-
**Triglycerides (mg/dL)**	0.23	0–0.002	0.02	**Triglycerides**	0.22	0.001–0.002	0.04
**Platelets (cells/mm**^**3**^**)**	0.37	0.001–0.004	<0.001	**Platelets**	0.36	0.001–0.004	<0.001
**White blood cells(cells/mm**^**3**^**)**	0.19	-0.004–0.096	0.07	**White blood cells**	-0.01	-0.056–0.051	0.92
**Child Pugh (A = 1; B = 2; C = 3)**	-	-	-	**Child Pugh**	-	-	-
**Spleen size (cm**^**2**^**)**	-	-	-	**Spleen size**	-	-	-
**Varices (No = 0; Yes = 1)**	-	-	-	**Varices**	-	-	-
**CRYPTOPGENIC CIRRHOSIS**							
**Univariate**				**Multivariate**			
	**β**	**95% CI for B**	**p**		**β**	**95% CI for B**	**p**
**Age (years)**	0.05	-0.01–0.01	0.7	**Age**	-	-	-
**Sex (F = 0; M = 1)**	-0.12	-0.21–0.08	0.36	**Sex**	-	-	-
**AST (IU/L)**	-0.16	-0.007–0.002	0.23	**AST**	-	-	-
**ALT (IU/L)**	-0.19	-0.01–0.001	0.13	**ALT**	-	-	-
**Total Cholesterol (mg/dL)**	-0.04	-0.002–0.002	0.74	**Total Cholesterol**	-	-	-
**LDL Cholesterol (mg/dL)**	-0.12	-0.003–0.001	0.34	**LDL Cholesterol**	-	-	-
**HDL Cholesterol (mg/dL)**	-0.01	-0.01–0.01	0.93	**HDL Cholesterol**	-	-	-
**Triglycerides (mg/dL)**	0.17	0–0.002	0.18	**Triglycerides**	-	-	-
**Platelets (cells/mm**^**3**^**)**	0.30	0–0.003	0.02	**Platelets**	0.18	-0.001–0.003	0.21
**White blood cells (cells/mm**^**3**^**)**	0.31	0.01–0.10	0.01	**White blood cells**	0.20	-0.2–0.09	0.17
**Child Pugh (A = 1; B = 2; C = 3)**	0.01	-0.11–0.12	0.93	**Child Pugh**	-	-	-
**Spleen size (cm**^**2**^**)**	-0.20	-0.004–0.001	0.13	**Spleen size**	-	-	-
**Varices (No = 0; Yes = 1)**	-0.11	-0.21–0.09	0.43	**Varices**	-	-	-
**CIRRHOSIS OF KNOWN ETIOLOGY**							
**Univariate**				**Multivariate**			
	**β**	**95% CI for B**	**p**		**β**	**95% CI for B**	**p**
**Age (years)**	0.08	-0.004–0.01	0.46	**Age**	-	-	-
**Sex (F = 0; M = 1)**	0.19	-0.02–0.26	0.08	**Sex**	0.25	0.053–0.281	0.005
**AST (IU/L)**	-0.08	-0.003–0.002	0.50	**AST**	-	-	-
**ALT (IU/L)**	-0.03	-0.002–0.001	0.82	**ALT**	-	-	-
**Total Cholesterol (mg/dL)**	0.22	0–0.003	0.04	**Total Cholesterol**	-0.019	-0.094–0.926	0.49
**LDL Cholesterol (mg/dL)**	0.20	0–0.004	0.07	**LDL Cholesterol**	-0.078	-0.005–0.003	0.70
**HDL Cholesterol (mg/dL)**	0.06	-0.003–0.005	0.57	**HDL Cholesterol**	-	-	-
**Triglycerides (mg/dL)**	0.03	-0.001–0.002	0.82	**Triglycerides**	-	-	-
**Platelets (cells/mm**^**3**^**)**	0.58	0.002–0.004	<0.001	**Platelets**	0.55	0.002–0.004	<0.001
**White blood cells (cells/mm**^**3**^**)**	0.52	0.04–0.08	<0.001	**White blood cells**	0.34	0.017–0.058	0.001
**Child Pugh (A = 1; B = 2; C = 3)**	-0.11	-0.18–0.06	0.32	**Child Pugh**	-	-	-
**Spleen size (cm**^**2**^**)**	-0.26	-0.005–0.002	0.02	**Spleen size**	0.11	-0.001–0.003	0.37
**Varices (No = 0; Yes = 1)**	-0.21	-0.218–0.004	0.06	**Varices**	-0.01	-0.097–0.091	0.96
**CIRRHOTIC POPULATION AS A WHOLE**							
**Univariate**				**Multivariate**			
	**β**	**95% CI for B**	**p**		**β**	**95% CI for B**	**p**
**Age (years)**	0.07	-0.003–0.007	0.40	**Age**	-	-	-
**Sex (F = 0; M = 1)**	0.07	-0.06–0.14	0.41	**Sex**	-	-	-
**AST (IU/L)**	-0.09	-0.003–0.001	0.27	**AST**	-	-	-
**ALT (IU/L)**	-0.06	-0.002–0.001	0.47	**ALT**	-	-	-
**Total Cholesterol (mg/dL)**	0.13	0.001–0.002	0.11	**Total Cholesterol**	-	-	-
**LDL Cholesterol (mg/dL)**	0.08	-0.001–0.002	0.36	**LDL Cholesterol**	-	-	-
**HDL Cholesterol (mg/dL)**	0.04	-0.002–0.003	0.66	**HDL Cholesterol**	-	-	-
**Triglycerides (mg/dL)**	0.09	0.0001–0.001	0.27	**Triglycerides**	-	-	-
**Platelets (cells/mm**^**3**^**)**	0.50	0.002–0.003	<0.001	**Platelets**	0.41	0.001–0.003	<0.001
**White blood cells (cells/mm**^**3**^**)**	0.45	0.04–0.07	<0.001	**White blood cells**	0.30	0.02–0.06	<0.001
**Child Pugh (A = 1; B = 2; C = 3)**	-0.06	-0.11–0.05	0.47	**Child Pugh**	-	-	-
**Spleen size (cm**^**2**^**)**	-0.22	-0.004-(-0.001)	0.01	**Spleen size**	0.07	-0.001–0.002	0.41
**Varices (No = 0; Yes = 1)**	-0.17	-0.18-(-0.005)	0.04	**Varices**	-0.01	-0.08–0.08	0.94

As reported in Table 3, in the cirrhotic population as a whole, age, liver enzymes and Child-Pugh class were not significantly different when compared according to different levels of LAL activity (normal, mildly reduced, severely reduced).

**Table 3:** Main characteristics of the whole cirrhotic population according to different levels of LAL activity (normal, mildly reduced and severely reduced).

	LAL activity (nmol/spot/h)			
	Normal(≥0.8)	Mildly reduced (0.4≤LAL<0.8)	Severely reduced (<0.4)	p
**n. of patients**	40	84	27	-
**Age (years)**	67.5 ± 9.1	66.1 ± 10.7	65.7 ± 10.6	0.4
**Sex (M/F) (%)**	23/17 (58%/42%)	51/33 (61%/39%)	11/16 (41%/59%)	0.2
**AST (IU/L)**	38.5 ± 22.8	43.8 ± 29.7	43.7 ± 22.9	0.6
**ALT (IU/L)**	40.3 ± 29.4	45.4 ± 40.7	40.4 ± 25.3	0.8
**Total cholesterol (mg/dL)**	154.4 ± 43.8	148.3 ± 39.7	137.1 ± 42.2	0.5
**HDL cholesterol (mg/dL)**	43.9 ± 22.4	43.1 ± 15.7	45.4 ± 16.3	0.7
**LDL cholesterol (mg/dL)**	83.6 ± 36.5	81.1 ± 33.1	76.8 ± 25.0	0.9
**Triglycerides (mg/dL)**	134.3 ± 78.3	120.6 ± 49.2	105.0 ± 44.5	0.4
**White blood cells (cells/mm**^**3**^**)**	6690 ± 3541	5000 ± 1788	4356 ± 1557	<0.001
**Platelets (cells/mm**^**3**^**)**	154825± 74932	109678±53558	92222 ± 48157	<0.001
**Child-Pugh class (A/B/C)(%)**	25/13/2 (63%/32%/5%)	49/28/7 (58%/33%/9%)	14/9/4 (52%/33%/15%)	0.6
**Oesophageal varices (Y/N)**	14/26	40/44 (48%/52%)	13/14	0.04
**(%)**	(35%/65%)	(35%/65%)	(48%/52%)	
**Splenic volume (cm**^**2**^**)**	54.1 ± 23.7	72.8 ± 32.7	70.3 ± 38.5	0.01

The univariate and multivariate regression analyses to LAL activity in the entire study population are reported in Table 4. As expected, being LAL activity significantly reduced in cirrhotic patients, in which liver enzymes are elevated and lipid parameters reduced with respect to healthy controls due to hepatic damage and malfunction, univariate analyses disclosed significant inverse associations between LAL activity and transaminase levels, and significantly direct ones between LAL activity and lipid parameters. Moreover, strongly significant direct associations were confirmed between white blood cell and platelet numbers and LAL function. Notably, in the multivariate linear regression model, only white blood cells, platelets and the condition of cirrhosis maintained significant independent associations with LAL function, while an inverse association between LAL activity and LDL cholesterol was at the limit of statistical significance.

**Table 4:** Univariate and multivariate linear regression analysis to LAL activity in the entire study population.

Univariate				Multivariate			
	β	95% CI for B	p		β	95% CI for B	p
**Age (years)**	0.02	-0.004–0.006	0.77	**Age**	-	-	-
**Sex (F = 0; M = 1)**	-0.04	-0.13–0.06	0.50	**Sex**	-	-	-
**AST (IU/L)**	-0.28	-0.007-(-0.003)	<0.001	**AST**	-0.02	-0.003–0.003	0.83
**ALT (IU/L)**	-0.17	-0.004-(-0.001)	0.008	**ALT**	-0.03	-0.003–0.002	0.77
**Total Cholesterol (mg/dL)**	0.3	0.001–0.003	<0.001	Total Cholesterol*	-	-	-
**LDL Cholesterol (mg/dL)**	0.25	0.001–0.004	<0.001	**LDL Cholesterol**	-0.12	-0.002–0.001	0.05
**HDL Cholesterol (mg/dL)**	0.12	0.001–0.006	0.07	**HDL Cholesterol**	0.02	-0.002–0.003	0.75
**Triglycerides (mg/dL)**	0.16	0.001–0.002	0.01	**Triglycerides**	0.12	0.0001–0.001	0.17
**Platelets (cells/mm**^**3**^**)**	0.60	0.002–0.003	<0.001	**Platelets**	0.44	0.001–0.003	<0.001
**White blood cells (cells/mm**^**3**^**)**	0.40	0.05–0.10	<0.001	**White blood cells**	0.18	0.011–0.052	0.003
**Cirrhosis (No = 0; Yes = 1)**	-0.48	-0.48-(-0.30)	<0.001	**Cirrhosis**	-0.16	-0.25-(-0.005)	0.04

*excluded from the final model due to collinearity

### Results of the analysis for E8SJM mutation in the LIPA gene

Among cirrhotic patients and healthy controls, 111 subjects showed a LAL activity <0.8 nmol/spot/h and were studied for the rs116928232 c.894G>A mutation. Only 1 patients with HCV-related cirrhosis, but none of other cirrhotics or healthy subjects, was found heterozygous for the E8SJM mutation.

## Discussion

The present study demonstrates, for the first time, that liver cirrhosis from any etiology is characterized by a significant reduction of LAL activity, which is likely on an acquired base and independent from the etiology of hepatic disease. Future investigations are needed to identify all the determinants of LAL activity reduction in cirrhosis, as well as its possible physiopathological consequences.

LAL-d is a rare autosomal recessive disease characterized by profound alterations in intracellular trafficking of cholesterol and triglycerides. In Wolman disease, which is caused by the most severe alterations affecting the LIPA gene (nonsense mutations, frameshift defects, ect…), patients typically develop symptoms at 2–4 months of age and usually die before age 12 months [14,15]. CESD, which is the milder variant, is determined by mutations permitting a minimal residual LAL activity. Mean age at symptom onset is 5 years but cases diagnosed as late as at 44 years in men and at 68 years in women have been reported [3]. The clinical presentation of CESD is highly heterogeneous and its diagnosis is frequently challenging, being possibly misdiagnosed as a disorder of lipid metabolism or as NAFLD, NASH and cryptogenic cirrhosis [5,15–17].

Liver involvement is almost ubiquitous in Wolman disease and CESD, and patients with cryptogenic liver disease are certainly among the categories to be screened for LAL-d [1]. Based on this background, our study was specifically designed to evaluate DBS-determined LAL activity in a population of patients with cryptogenic liver cirrhosis. Moreover, in order to distinguish between the hereditary *Vs* the acquired nature of the eventual reduction of LAL activity, we decided to include a control group of patients with cirrhosis of known etiology and, mainly, to screen all subjects with a LAL activity <0.8 nmol/spot/h for the E8SJM mutation in the LIPA gene. The cut-off value of 0.8 nmol/spot/h was chosen to guarantee the maximal assay sensitivity, since LAL-d patients (homozygous) are known to show a LAL activity <0.03 nmol/spot/h, while carriers (heterozygous) between 0.15 and 0.4 nmol/spot/h [8]. Moreover, in order to evaluate the contribution of genetics on the biochemical decrease of LAL activity, we also performed the analysis of the E8SJM mutation, which is responsible for 50–70% of LAL-d cases [2,3]. Actually, the screening of the E8SJM mutation has been commonly applied in studies of prevalence [18], and also when testing the contribution of a reduced LAL activity on the risk of dyslipidemia, myocardial infarction or coronary artery disease [19].

Our results demonstrate that the DBS-determined LAL activity is severely reduced in patients with cryptogenic cirrhosis and that this reduction is not secondary to the inclusion of any subject carrying the E8SJM mutation in the LIPA gene either at the homozygous or at the heterozygous level. Indeed, a comparable LAL activity reduction was observed in patients with cirrhosis of known etiology, substantiating the hypothesis that LAL function is impaired on an acquired base, likely associated with or determined by the condition of cirrhosis.

When interpreting these results, the clinical characteristics of our population of cryptogenic cirrhotics should be carefully taken into account. Indeed, mean BMI of these patients was in the range of obesity (30.9 ± 7.8 kg/m^2^), and 68% of them had type 2 diabetes, both conditions which are strongly associated with NASH. One may comment that the ideal population to screen for possible LAL-d is that of cryptogenic cirrhotics who lack a dysmetabolic background. However, inclusion/exclusion criteria for selecting such a group of patients are almost impossible to draw up due to: 1) the possibility that overweight/obesity disappear once cirrhosis has developed; 2) the fact that type 2 diabetes is a well-known complications of cirrhosis of any etiology. Moreover, based on the recent finding of a reduced LAL activity in patients with NAFLD and NASH [6], the present study had not the purpose to categorically rule out cases of cirrhosis possibly representing the evolution of NASH.

As expected, since most of the enzymatic activity dosed in DBS derives from leukocytes [9,10], the lower white blood cell count observed in cirrhotic patients is partially responsible for the reduction of DBS-determined LAL activity in this population. Indeed, when using DBS, enzymatic activity is normalized for the diameter of the spot and not for protein concentration [9]. Therefore, although to date this problem has not been specifically acknowledged in literature, the concentration of white blood cells per mm^3^ was to be expected and is here demonstrated to affect the result of DBS-determined LAL activity. Interestingly, LAL activity reduction in cirrhosis was found to be affected even more strongly by the reduction of platelet number. Indeed, the association between LAL function and platelets was tighter than that observed with white blood cells and with other surrogate indices of portal hypertension, such as splenic volume and presence of oesophageal varices, and it was observed also in the population of healthy controls. Actually, although platelets are known to contain lysosomes [20], these results were unexpected since the contribution of platelets when determining lysosomal enzyme activity in DBS has been never specifically addressed.

Nevertheless, the condition of cirrhosis was found to affect LAL function independently from the reduction of white blood cell and platelet counts. The availability of different substrates, in particular LDL cholesterol, and many other factors, i.e., glucagon, estradiol, thyroid hormones, etc., have been shown to modulate LAL activity *in vitro* [21]. Consistently, at the multivariate analysis in the entire study population, an inverse association at the limit of statistical significance between LAL activity and LDL cholesterol was evidenced. Conversely, LAL activity was not correlated with Child-Pugh class, suggesting that, whatever the mechanisms impacting LAL activity during cirrhosis, they are not tightly dependent on liver function. Future investigations should be certainly aimed to understand which factor is mostly responsible for the reduced LAL function observed in cirrhosis.

Even more interesting will be trying to individuate if the reduced LAL function is responsible for any complication concerning liver damage and/or extrahepatic manifestations of cirrhosis. Differently from what observed by Baratta *et al*. in NAFLD patients [6], we didn’t find any independent association between LAL activity and transaminase levels. However, clearly, this finding is not sufficient to definitely rule out a possible contribution of the reduced LAL function to liver damage in cirrhosis. Due to the broad expression of LAL [4], which has a key role in cellular cholesterol metabolism, an impaired LAL function may be responsible also of different extrahepatic complications of cirrhosis, such as adrenal insufficiency, primary hypogonadism, macrocytosis, etc… [22–24]. In this context, it should be noted that Wolman disease is the cause of 3% of cases of primary adrenal insufficiency in children [25]. Nevertheless, concerning this point, one important issue should be taken into account. Here LAL activity was determined in DBS by the new method which has been recently introduced and validated by Hamilton *et al*. [8]. This blood test for LAL demonstrated excellent differentiation between healthy subjects, carriers and affected individuals, and, also due to its simplicity, it has replaced previous techniques based on measuring enzyme activity in cultured fibroblast, peripheral leucocytes or liver tissue [1]. However, any test applied to determine LAL activity was developed to screen for the genetic disease, in which LAL function is equally affected in all cells and tissues. Conversely, in all the conditions in which LAL activity is impaired due to epigenetic/post-transcriptional factors, we can’t be sure that it is affected the same way throughout the body. Future investigations should be certainly aimed to compare peripheral and hepatic LAL activity in NAFLD and in liver cirrhosis, and animal models would certainly contribute to clarify this point. In conclusion, the present results are the first to demonstrate a significant acquired reduction of LAL activity in patients with cryptogenic cirrhosis and with cirrhosis of known etiology. Considering the profound impact of an altered LAL function on intracellular cholesterol pathways, the significant disorders commonly observed during cirrhosis which derive from an altered cholesterol metabolism and the current availability of a LAL replacement treatment, further experimental and human research is awaited to clarify rapidly the precise causes and consequences of impaired LAL function observed in cirrhosis. The data hereby reported are also the first to raise concerns about the possibility that DBS-determined lysosomal enzyme activities may be affected by white blood cell and platelet counts, and claim to further studies aimed to verify the specificity of these tests when applied in determined patient populations, such as cirrhotics.

## Supporting Information

S1 File Individual data database. (XLSX)
